# Activation‐Induced Turbostratic Ordering in pDCPD‐Derived Hard Carbon Enables Stable Carbonate‐Based Sodium‐Ion Storage

**DOI:** 10.1002/advs.78311

**Published:** 2026-10-18

**Authors:** David Schuster, Glen J. Smales, Max Schmallegger, Julian M Rosalie, Konstantin Kraushaar, Andreas Lißner, Julia Bohdal, Pia Winter, Bernd Fuchsbichler, Stefan Koller, Sebastijan Kovačič, Christian Slugovc

**Affiliations:** ^1^ Christian Doppler Laboratory for Organocatalysis in Polymerization Graz University of Technology Graz Austria; ^2^ VARTA Innovation GmbH Graz Austria; ^3^ Institute for Chemistry and Technology of Materials Graz University of Technology Graz Austria; ^4^ Institute of Inorganic Chemistry Graz University of Technology Graz Austria; ^5^ Institute for Physical and Theoretical Chemistry Graz University of Technology Graz Austria; ^6^ Federal Institute of Materials Research and Testing (BAM) Berlin Germany; ^7^ Institute of Inorganic Chemistry TU Bergakademie Freiberg Freiberg Germany; ^8^ Institute of Physical Chemistry TU Bergakademie Freiberg Freiberg Germany; ^9^ Catalysis and Organic Synthesis Research Group, Faculty of Chemistry and Chemical Engineering University of Maribor Maribor Slovenia; ^10^ Department of Inorganic Chemistry and Technology National Institute of Chemistry Ljubljana Slovenia

**Keywords:** ${\mathrm{CO}}_{2}$ activation, cycle stability, hard carbon, poly(dicyclopentadiene), sodium–ion batteries

## Abstract

Sodium–ion batteries are widely considered a promising alternative to lithium‐ion batteries, due to the abundance and low cost of their raw materials. However, the development of high‐performance and cost‐effective electrode materials remains a significant challenge, particularly when competing with lithium iron phosphate systems. Herein, we present a hard carbon material derived from readily available poly(dicyclopentadiene) for use as a negative electrode in SIBs. This study investigates the influence of carbonization temperature and subsequent ${\mathrm{CO}}_{2}$ activation on the hard carbon structure and electrochemical performance. The impact of activation proved to be dependent upon the first stage carbonization temperature, creating greater local ordering for samples carbonized at higher temperatures. The removal of defects and increase in interlayer distance resulted in a turbostratic structure that enhanced rate performance and capacity retention when compared with non‐activated samples. The optimized hard carbon exhibits a reversible capacity of 352 mAh $g^{-1}$ at 0.1 C and a capacity retention of 88.1% after 500 cycles with a carbonate based electrolyte. This work introduces a cost‐effective approach for producing hard carbon materials with distinctive nanostructure and provides valuable insights into achieving long cycle life in sodium–ion batteries.

## Graphical abstract

Hard carbon derived from templated poly(dicyclopentadiene) as anode for sodium–ion batteries was engineered through carbonization and second‐step ${\mathrm{CO}}_{2}$ activation. High‐temperature carbonization and activation increased local ordering while reducing defects without inducing structural fracture. The resulting turbostratic carbon delivered enhanced rate capability and long‐term cycling stability, achieving 352 mAh $g^{-1}$ and 88.1% capacity retention after 500 cycles in full‐cell configuration.

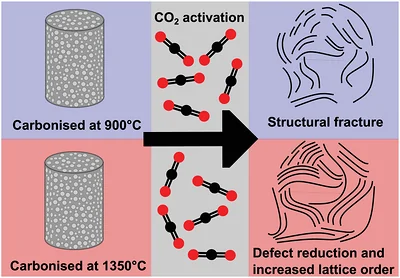

## 1 Introduction

Regardless of the chosen pathway for energy generation, the growing demand for electrical energy storage remains a fundamental challenge for modern societies. Global battery storage capacity is projected to increase from 27 GW, in 2021, to 1000–3500 GW by 2050 [1]. To address the challenges associated with increasing production capacity, fluctuating lithium prices, uneven global resource distribution, and the environmental impact of mining, alternatives to market‐leading lithium–ion battery (LIB) systems are actively being researched [2, 3, 4, 5, 6]. One promising alternative is sodium‐ion batteries (SIBs). SIBs have the potential to be more cost‐efficient due to the approximately 1000‐fold higher abundance of sodium in the Earth's crust compared to lithium. As SIBs possess many similarities to LIBs, the majority of materials can be manufactured and processed using equipment already in place for LIB. Another key advantage is that aluminium, which is both cheaper and lighter than copper, can also be used as the current collector for the negative electrode [7, 8, 9, 10, 11]. However, the large‐scale commercialization of SIBs has been hindered by direct competition with LIBs, especially those employing lithium iron phosphate (LFP) cathodes, which offer comparable performance to SIBs, with both avoiding the use of critical or expensive transition metals, such as cobalt or nickel [12, 13, 14, 15, 16, 17]. In contrast, SIBs often rely on comparatively expensive Ni‐rich layered transition metal oxides (LTOs) or alternative materials such as Prussian blue/white analogues and ${\mathrm{Na}}_{4}{\mathrm{Fe}}_{3}{\left({\mathrm{PO}}_{4}\right)}_{2}P_{2}O_{7}$ (NFPP), which have attracted industrial interest due to their cobalt‐ and nickel‐free compositions, although challenges related to cost, synthesis complexity, and large‐scale production remain. These compounds present significant challenges for large‐scale production, particularly when transitioning from simple dye precursors to electrochemically active cathode materials [18]. A similar trend is observed with negative electrode materials, where hard carbon (HC) dominates the literature [19, 20, 21, 22]. High‐performance hard carbons are frequently derived from carbonized biomass precursors such as peanut shells, carbohydrates, starch, or phenolic resins. During pyrolysis, carbon layers develop disordered microstructures consisting of nanopores, turbostratic graphitic domains, and surface defects. These hard carbon anodes often show limited capacity retention after hundreds of cycles in industry‐standard carbonate electrolytes, however. Studies have shown that without special additives or an engineered interphase, carbonate systems exhibit accelerated impedance growth and reduced cycle life, often leading to a modest capacity retention (CR) of 70%–80% after 400–500 cycles [23, 24, 25, 26, 27, 28, 29].

These factors counteract the intrinsic cost advantage of sodium resources and highlight the need to develop cost‐effective cell concepts based on readily available materials for all battery components.

One such potential precursor is dicyclopentadiene (DCPD), which due to its widespread availability and high carbon content, represents a promising precursor for hard carbon anode materials in SIBs [30].

In previous work, our group reported on the synthesis, internal structure, and mechanical properties of polydicyclopentadiene (pDCPD) foams prepared from high internal phase emulsions (HIPEs) of water in dicyclopentadiene (DCPD) [31, 32, 33]. Subsequent pyrolysis of these polyHIPE structures enabled their application as carbon current collectors in Li‐$O_{2}$ systems, as well as cathode materials in Li‐S batteries, as demonstrated in preliminary studies. Notably, controlled oxidation at 40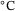 of the porous polyHIPE template prior to carbonization allowed the macroscopic structure to be retained, resulting in the formation of mechanically stable hard carbon architectures [34, 35]. This pre‐oxidative treatment preserves the three‐dimensional porous framework during carbonization, yielding a conductive hard carbon material with a (macro)porosity‐retaining morphology and carbon yields of 36%–38%. In this current work, we systematically investigate the influence of carbonization temperature and subsequent ${\mathrm{CO}}_{2}$ activation on the structural evolution and electrochemical performance of the resulting hard carbon as a negative electrode material for sodium–ion batteries. Particular emphasis is placed on correlating heat‐treatment conditions with microstructural ordering, defect density, porosity, and presenting the influence on electrode performance. By tailoring the carbonization and activation procedure, this study aims to establish pDCPD‐derived hard carbons as a versatile and scalable anode matrix for sodium–ion energy storage systems.

## 2 Results and Discussion

### 2.1 Structural Analysis

Synthesis of the polyHIPEs was carried out according to procedures in the literature [34]. With the addition of a mild oxidation step (40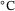 for seven days) the carbonized structure is maintained much more readily with significantly higher yields (36%–38%) of conductive carbon, when compared to carbonization without this mild‐oxidation step (12%–13%). To systematically investigate the influence of pyrolysis temperature and the effect of ${\mathrm{CO}}_{2}$ activation, two sets of samples were produced using identical heating procedures, with a final activation step performed in ${\mathrm{CO}}_{2}$ or $N_{2}$ atmospheres to produce activated and non‐activated materials.

Figure 1 shows how these procedures were carried out. The first plateau was applied to carbonize and rearrange the pDCPD structure into a hard carbon structure with differently distributed nanopore sizes and interlayer distances. This was performed with four different plateau/upper temperatures (900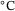, 1050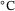, 1200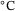, and 1350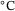) held for two hours. The second plateau or activation step at 900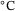, was performed for removal of deficiencies in the structure according to the Boudouard equilibrium (Equation 1).

(1) $$CO_{2}\left(g\right)+C\left(s\right)⇌2\;CO\left(g\right)$$

**FIGURE 1:**
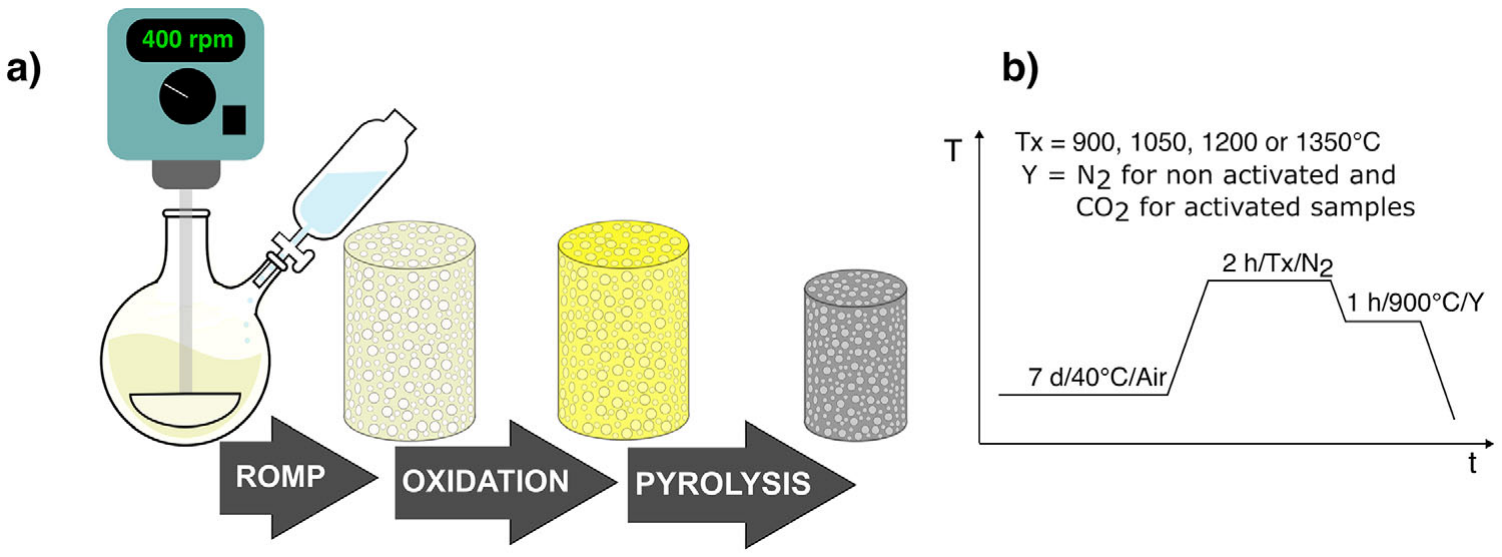
(a) Depiction of hard carbon synthesis. (b) Illustration of heat treatment and atmospheric conditions for different hard carbon samples.

To ensure comparability between processes, the non‐activated samples were treated with a second plateau under an inert $N_{2}$ atmosphere too, using the same conditions as ${\mathrm{CO}}_{2}$ activated samples.

Ball milling of the hard carbon material resulted in edged chunks with particle size distribution and exemplary images provided in Figures S1 and S2. During the carbonization, the polyHIPE foam structure was maintained, as depicted in Figure S3, which shows sample HC1350_CO2. To further investigate the influence of ${\mathrm{CO}}_{2}$ activation, samples heated to 900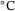 and 1350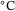 were examined using TEM (Figure 2). As clearly visible in Figure 2, the non‐activated samples, HC900 and HC1350, exhibit an amorphous structure with few distinguishable features. Electron diffraction analysis indicates that HC900 exhibits a higher degree of order compared to its ${\mathrm{CO}}_{2}$‐treated counterpart. Interestingly, the activated sample HC900_CO2 exhibits a disrupted structure, possibly resulting from stress induced by chemical reactions occurring during ${\mathrm{CO}}_{2}$ activation. Furthermore, the electron diffraction pattern shows reduced long‐range order compared to the non‐activated samples. In contrast, ${\mathrm{CO}}_{2}$ activation resulted in increased structural order for sample HC1350_CO2, as evidenced by electron diffraction. In addition, a disordered layered structure, typical for hard carbon materials, is much more visible for this sample (see Figure S4). The differing effects of ${\mathrm{CO}}_{2}$ activation on samples carbonized at 900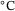 and 1350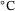 may be explained by structural changes occurring at temperatures above 900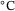. This is first indicated by differences in mass loss during activation: ${\mathrm{CO}}_{2}$ activation of HC900 resulted in an additional mass loss of 11%, whereas the mass loss associated with the formation of HC1350_CO2 amounts to only about 2% when compared to the corresponding non‐activated samples. These structural differences are further reflected in the $N_{2}$ adsorption–desorption isotherms shown in Figure S5, which were used to determine the Brunauer–Emmett–Teller (BET) specific surface area ($S_{\mathrm{BET}}$) of the various hard carbon samples. Non‐activated samples exhibit relatively low surface areas ranging from 0.9 to 5.0 $m^{2}$ $g^{-1}$. In contrast, ${\mathrm{CO}}_{2}$ activation leads to a substantial increase in surface area for HC900_CO2 and HC1050_CO2, yielding $S_{\mathrm{BET}}$ values of 380.8 and 125.1 $m^{2}$ $g^{-1}$, respectively. For samples subjected to higher first‐stage carbonization temperatures, specifically 1200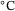 and 1350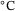, subsequent ${\mathrm{CO}}_{2}$ activation has little effect on the BET surface area, resulting in only slight increases to 7.7 and 18.5 $m^{2}$ $g^{-1}$.

**FIGURE 2:**
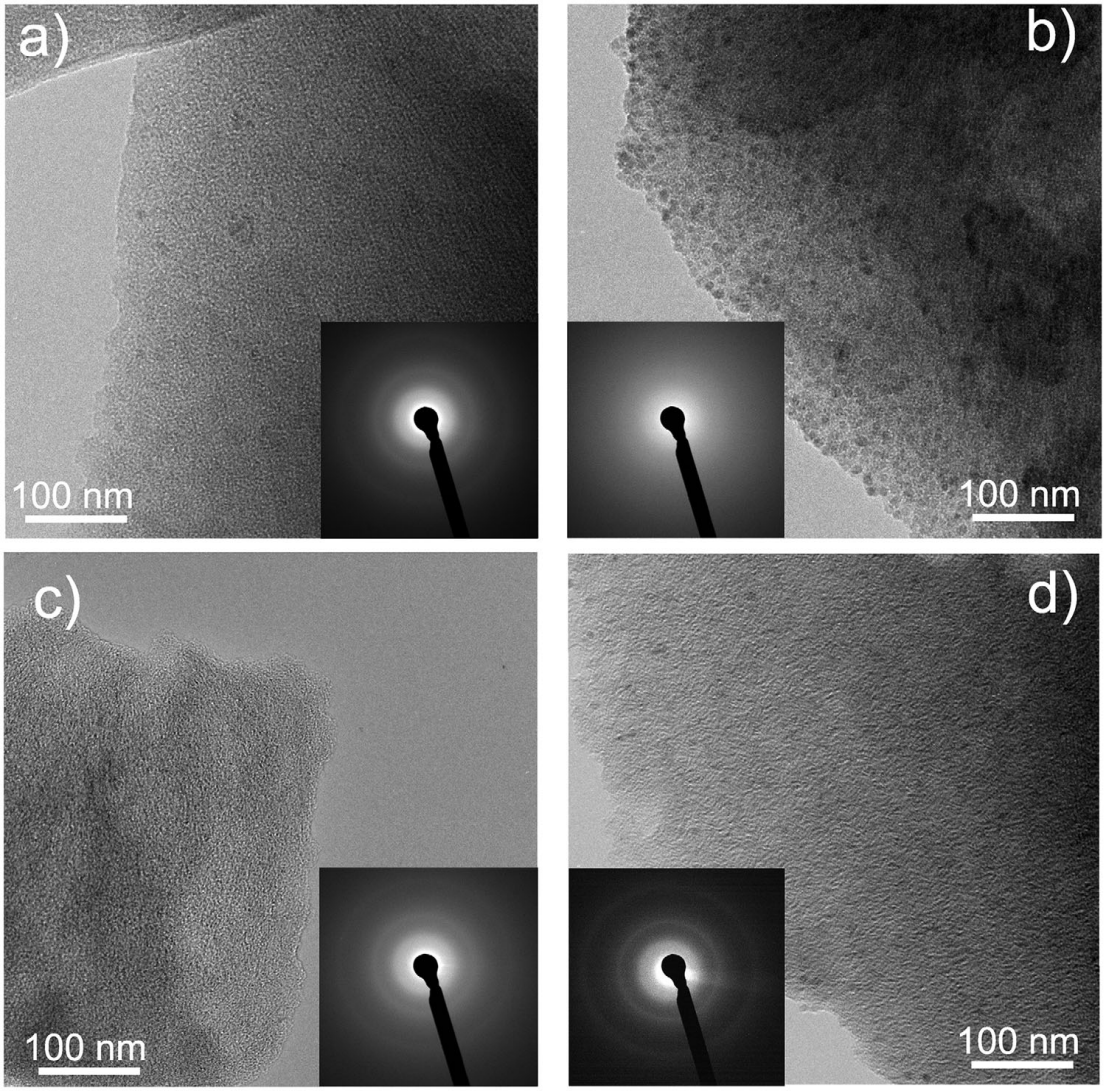
TEM image and corresponding diffraction of (a) HC900, (b) HC900_CO2, (c) HC1350 and (d) HC1350_CO2.

To further validate these findings, a second set of BET measurements was performed using Ar as the adsorbate gas. The same trend was observed under these conditions, with the activated samples carbonized at lower temperatures exhibiting the largest increase in specific surface area, reaching 80.4 and 69.9 $m^{2}$ $g^{-1}$ for HC900_CO2 and HC1050_CO2, respectively. In contrast, samples carbonized at higher temperatures showed substantially smaller increases, with specific surface areas of 20.5 and 11.7 $m^{2}$ $g^{-1}$ for HC1200_CO2 and HC1350_CO2, respectively. The corresponding data can be seen in Figure S5b. These observations further corroborate the conclusion that the resistance of the material to second‐stage activation increases with higher first‐stage carbonization temperatures, this stands in agreement with the TEM results. Elemental analysis supports this interpretation further, indicating a higher carbon content for samples carbonized at 1350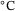 relative to those carbonized at 900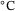. Following ${\mathrm{CO}}_{2}$ activation, a reduction in carbon content is observed, which can be attributed to carbon removal via the Boudouard reaction (Table 1).

**TABLE 1:** Physical parameters of hard carbon samples. *d002 distances given for data obtained via WAXS (${\mathrm{WA}}_{002}$), WAXS with added Si reference (${\mathrm{Si}}_{002}$), and fitted from the SAXS pattern (${\mathrm{SA}}_{002}$). Elemental analysis data (EA) given in wt.%. **$N_{2}$ and Ar BET specific surface areas. Samples denoted as ”n.d.” were not determined from Ar adsorption because the Ar uptake was insufficient to obtain a reliable BET fit.

Sample	Pore size [nm]	$WA_{002}/Si_{002}/SA_{002}$* [Å]	$I_{D}/I_{G}$	$S_{\mathrm{BET}}\;N_{2}/\mathrm{Ar}$** [$m^{2}$ $g^{-1}$]	${\mathrm{EA}}_{H}$	${\mathrm{EA}}_{N}$	${\mathrm{EA}}_{O}$	${\mathrm{EA}}_{C}$
HC900	1.65	3.82/3.84/3.80	1.11	1.5/n.d.	0.86%	0.10%	8.28%	90.76%
HC1050	1.79	3.74/3.78/3.72	1.35	0.9/n.d.	—	—	—	—
HC1200	2.04	3.71/3.72/3.69	1.51	5.0/4.1	—	—	—	—
HC1350	2.23	3.69/3.65/3.63	1.68	4.0/5.7	0.22%	<0.10%	0.50%	99.18%
HC900_CO2	1.23	3.83/3.88/3.82	1.21	380.8/80.4	1.10%	0.15%	12.60%	86.15%
HC1050_CO2	1.44	3.83/3.82/3.80	1.43	125.1/69.9	—	—	—	—
HC1200_CO2	1.63	3.81/3.80/3.78	1.65	7.7/20.5	—	—	—	—
HC1350_CO2	1.85	3.79/3.79/3.77	1.86	18.5/11.7	0.31%	0.15%	2.02%	97.52%

Wide‐angle X‐ray scattering (WAXS) patterns reveal a narrowing of the interlayer distance distribution with increasing plateau temperature (Figure 3a– c). Assuming that the number of graphene layers remains approximately constant, the resulting higher interlayer density in the sample carbonized at 1350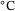 is likely to show enhanced resistance to stress induced by ${\mathrm{CO}}_{2}$ activation when compared to HC900. The higher mass loss observed for HC900 after ${\mathrm{CO}}_{2}$ activation may therefore be attributed to a higher defect density and increased reactivity, rendering it more susceptible to carbon gasification. In contrast, HC1350, with its more ordered and densely packed carbon layers, exhibits greater resistance to ${\mathrm{CO}}_{2}$ activation, resulting in significantly lower mass loss. The peak interlayer distances are listed in Table 1. Overall, the recorded WAXS patterns display broad peaks at approximately $23^{\circ }$ and $43^{\circ }$, corresponding to the (002) and (100) crystallographic planes of disordered carbon. Background‐subtracted patterns show a shift of the (002) peak toward higher angles with increasing heat‐treatment temperature, as shown in Figure S6.

**FIGURE 3:**
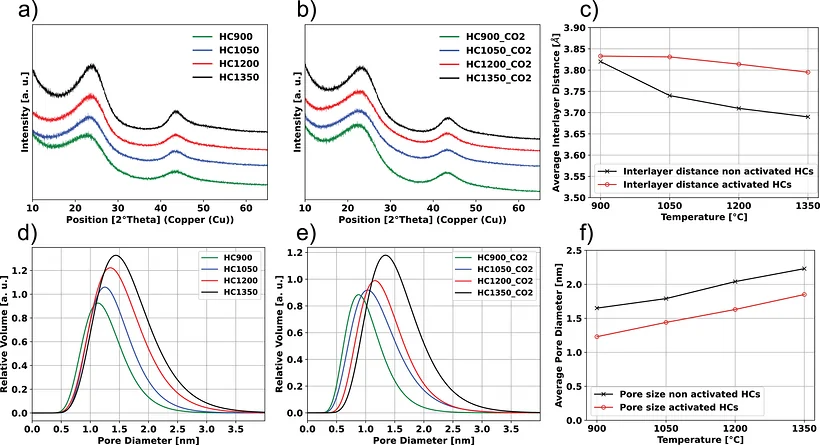
WAXS pattern of hard carbons for (a) non‐activated and (b) activated samples. (c) Average interlayer distance vs. temperature. Size distribution of pore diameter in hard carbons generated from SAXS data for (d) non‐activated and (e) activated samples. (f) Average pore diameter vs. temperature.

To further validate these findings, a Si reference was added to the hard carbon samples to account for possible $d_{002}$ peak shifting. The resulting patterns are illustrated in Figure S6b. Furthermore, small‐angle X‐ray scattering (SAXS) was applied to analyze the samples and the corresponding $d_{002}$ layer distribution fitted from the pattern as shown in Figure S7. The interlayer spacing obtained from spline fitted WAXS analysis, Si‐calibrated WAXS, and SAXS fitting shows the same trends, with differences of at most 0.06 Å, confirming the consistency of the applied methods. For the untreated hard carbons, $d_{002}$ decreases progressively with increasing carbonization temperature. In contrast, ${\mathrm{CO}}_{2}$ activation results in consistently larger interlayer spacings, ranging from approximately 3.83 to 3.79 Å, suggesting that activation induces a certain widening of the graphitic layers when compared to the untreated materials. All values are listed in Table 1.

SAXS was additionally employed to investigate the pore size distributions of the eight hard carbon samples. The pore size distributions obtained from fitted SAXS data are shown in Figure 3d,f. The data was derived using the exemplary fitting procedure provided in Figure S7. Increasing carbonization temperature results in a shift of the shoulder toward lower q values, indicating larger pore sizes, an effect frequently reported in the literature [36, 37, 38, 39]. The average pore diameter increases from 1.65 to 2.23 nm when comparing samples HC900 and HC1350. Interestingly, ${\mathrm{CO}}_{2}$ activation appears to slightly decrease the pore size when compared to the corresponding non‐activated samples. The volume‐weighted average pore diameter decreases to 1.23 nm for HC900_CO2 and to 1.85 nm for HC1350_CO2. This effect may arise from the slight increase in interlayer spacing, as widening of the graphitic layers could lead to shrinkage of the surrounding pores.

To further investigate the structural and chemical characteristics of the hard carbon samples, spectroscopic analyzes were performed using Raman spectroscopy and X‐ray photoelectron spectroscopy (XPS). The Raman spectra were deconvoluted to resolve the typically overlapping S, A, D, G and D2 bands, as commonly discussed in the literature [40, 41, 42, 43, 44]. The prominent D and G bands, observed at approximately 1330 and 1600 ${\mathrm{cm}}^{-1}$, correspond to disordered and ordered graphitic domains, respectively. Increasing the carbonization temperature leads to a decrease in the full width at half maximum (FWHM) of both bands, reflecting the progressive development of local short‐range structural ordering, in agreement with previous reports. Most notably, all ${\mathrm{CO}}_{2}$‐activated samples exhibit a diminished S band around 1200 ${\mathrm{cm}}^{-1}$ relative to their non‐activated counterparts. The S band is typically attributed to *sp* 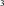 carbon bonds and $C_{7}$ 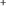 ring systems, and its reduction likely results from the removal of defective sites during ${\mathrm{CO}}_{2}$ activation, as previously observed by Xing et al. [45] Representative deconvoluted spectra for HC1350 and HC1350_CO2 are presented in Figure 4, while spectra for the remaining samples are shown in Figures S8 and S9. This interpretation is further supported by XPS measurements, which show a slight decrease in the *sp* 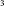 component for ${\mathrm{CO}}_{2}$‐activated samples. In contrast, the C–O related peaks in the C 1s spectra exhibit only minor variations. Complete XPS datasets are provided in Figures S10–S12 with quantitative values provided in Tables S1 and S2.

**FIGURE 4:**
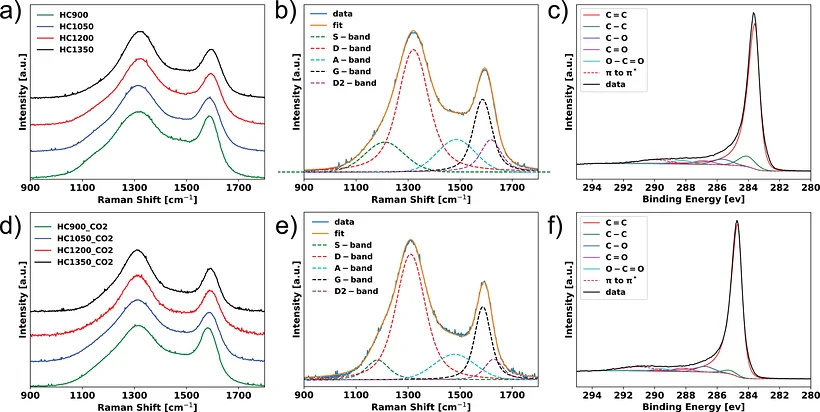
Raman spectra of (a) non‐activated and (d) activated hard carbon samples. Data were normalized and shifted on y‐axis for better illustration. Deconvoluted Raman spectra of (b) HC1350 and (e) HC1350_CO2. C1s XPS peak of (c) HC1350 and (f) HC1350_CO2.

In general, hard carbons with high local order are rarely reported in the literature. Cheng et al. synthesized a series of amorphous carbons spanning from relatively ordered soft carbons to highly disordered hard carbons, as well as intermediate “pseudo” materials that exhibited high overall disorder but pronounced local short‐range order [46]. As material HC1350_CO2 exhibits relatively high local order and turbostratic graphitic layers, but still features prominent nanopores and a significant plateau capacity (unusual for soft carbons), it might very well be described as a “pseudo‐hard carbon”.

### 2.2 Comparison of Electrochemical Performance as Sodium‐ion Anode Material

The influence of carbonization temperature and the sodium‐ion storage capability of the hard carbons was investigated via electrochemical testing in Swagelok half cells. The charge–discharge profiles of hard carbon samples prepared at various temperatures, activated and non‐activated, are depicted in Figure 5a–d. Remarkably, the sample produced at 900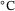 shows a predominantly sloping capacity, with little to no plateau region, a trend that is typically linked to the region below 100 mV. As carbonization temperature increases, the plateau region increases as well. This behavior can be attributed to the larger pore size, elucidated via SAXS analysis [43, 47, 48, 49]. The plateau and sloping capacities of all samples are shown in Figure 5e. As shown, three main trends can be observed. First, the specific capacity increases with increasing carbonization temperature. Second, the sloping capacity increases after ${\mathrm{CO}}_{2}$ activation but decreases with increasing temperature. Third, the plateau capacity increases with temperature and appears to be relatively unaffected by the ${\mathrm{CO}}_{2}$ activation.

**FIGURE 5:**
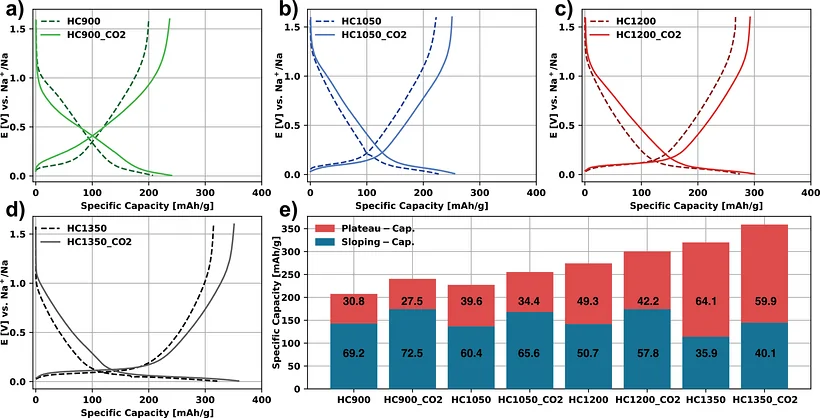
(a–d) Potential vs. specific capacity of second cycle at 0.1 C. Each hard carbon compared with corresponding activated sample. (e) Comparison of capacity below and above 100 mV for each sample with ratio of sloping/plateau capacity stated within bars.

Table 2 lists the initial coulombic efficiency (ICE) of all samples, which shows that ${\mathrm{CO}}_{2}$ treatment decreased the first cycle efficiency of all samples. The effect dominates for lower temperatures and is less severe for HC1350, as it decreases from 83.0% to 80.1% upon ${\mathrm{CO}}_{2}$ activation (HC1350_CO2). This can further be seen in the CV data depicted in Figure 6. For most samples, the irreversible capacity loss in the first cycle is seen in the sloping region at around 1.0–1.2 V during solid‐electrolyte interface (SEI) formation. Samples HC900_CO2 and HC1050_CO2, which underwent the largest change in $S_{\mathrm{BET}}$ surface area, exhibit a low ICE during galvanostatic cycling (58.0 and 62.7) and demonstrate significant losses in sloping and plateau regions, according to the CV profiles seen in Figure 6b,d.

**TABLE 2:** Electrochemical parameters of hard carbon samples. *Initial Coulombic efficiency. **Reversible capacity at 0.1 C. ***Average Coulombic efficiency over 140 cycles. ****Capacity retention after 140 cycles (after formation).

Sample	ICE* [%]	RC at 0.1 C** [mAh $g^{-1}$]	RC at 1 C [mAh $g^{-1}$]	CE*** [%]	CR**** [%]
HC900	73.3 ± 3.0	204	145	98.5 ± 0.7	63.0
HC1050	75.7 ± 3.4	222	111	98.8 ± 0.6	72.5
HC1200	79.9 ± 2.2	273	120	99.0 ± 0.5	71.6
HC1350	83.0 ± 2.0	323	149	99.2 ± 0.5	77.7
HC900_CO2	58.0 ± 2.5	240	173	99.9 ± 0.1	88.4
HC1050_CO2	62.7 ± 1.8	258	176	99.5 ± 0.2	86.1
HC1200_CO2	78.9 ± 2.5	300	187	99.5 ± 0.2	90.2
HC1350_CO2	80.1 ± 0.9	354	217	99.8 ± 0.1	91.1

**FIGURE 6:**
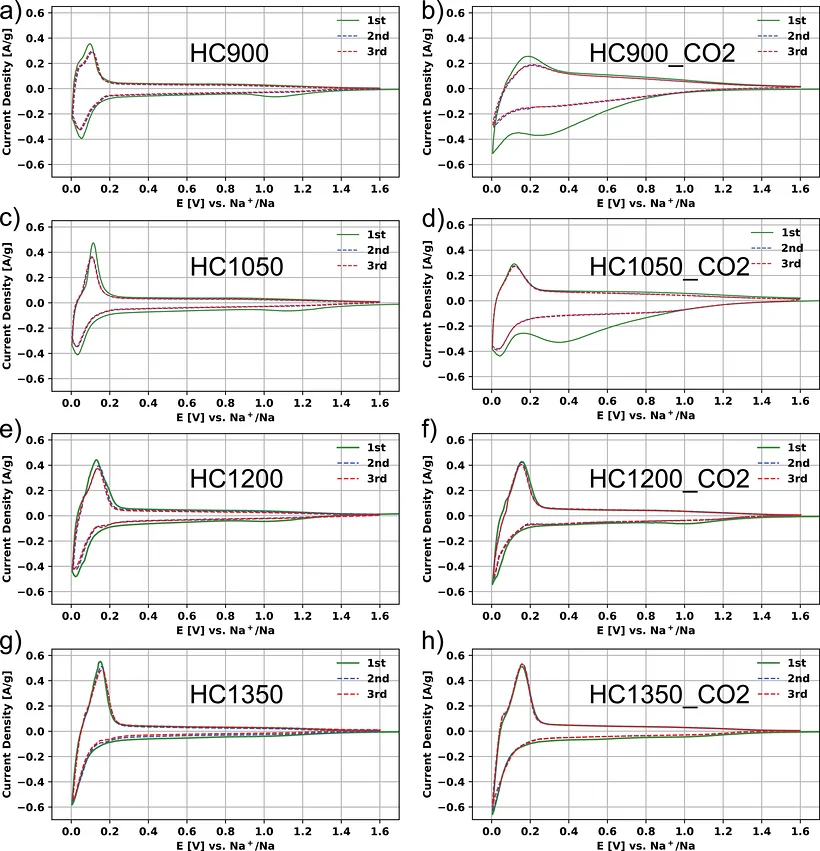
(a–h) Cyclic voltammograms of the first three cycles for each hard carbon sample at 30 μV/s from 1.6 V to 5 mV.

The electrochemical behavior was further investigated via testing the C‐rate performance. Here, cells were first formed via galvanostatic cycling at 0.1 C for 5 cycles, followed by rate tests from 0.1 to 2 C. For all rate capability tests, the C‐rate was defined based on the reversible specific capacity of each individual electrode, determined after five formation cycles at 0.1 C. This approach accounts for variations in capacity among different samples. Resulting charge capacities are provided in Figure 7a,b. Samples carbonized at lower temperatures show better rate performance, as the predominant sodium storage mechanism in these cases is attributed to adsorption, which is kinetically facile. Rate performance declines as carbonization temperature increases, aligning well with previous studies [38, 49]. The capacity decay at higher rates can mainly be attributed to a decrease in plateau capacity at higher C‐rates. It has been suggested that this effect might be due to significant Na‐ion trapping within pores or interlayers at high C‐rates during sodiation and desodiation resulting in capacity loss [50]. Interestingly, the ${\mathrm{CO}}_{2}$ activated samples show an improved rate performance when compared to their non activated counterparts, which can be linked to the increase in sloping capacity for all activated samples.

**FIGURE 7:**
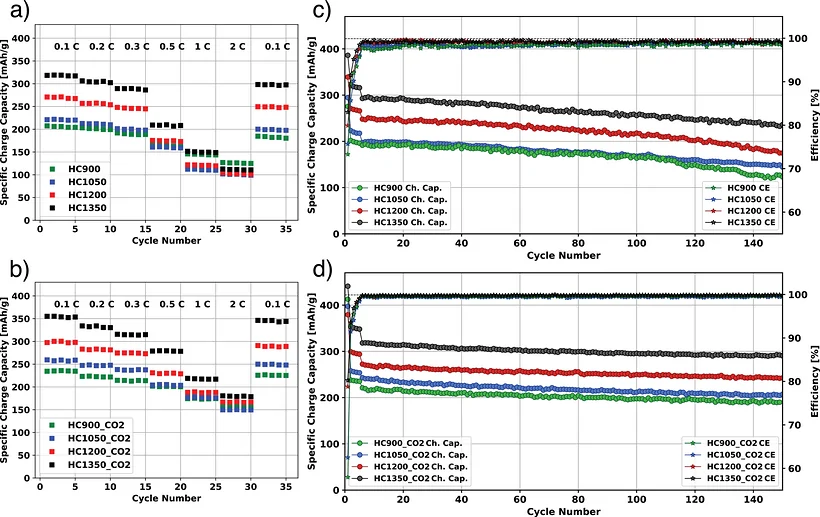
Rate performance tests for (a) non‐activated and (b) activated hard carbon samples. All cells were previously formed at 0.1 C for 5 cycles. Galvanostatic cycling data at 0.3 C for (c) non‐activated and (d) activated hard carbon samples. First 5 cycles show formation at 0.1 C.

As suggested by the WAXS/SAXS data (Table 1), ${\mathrm{CO}}_{2}$ treatment leads to higher average interlayer spacing, which might be responsible for the observed trend. While the surface area varies considerably with carbonization temperature after activation, the increase in sloping capacity is consistently observed for all activated samples. In contrast, all ${\mathrm{CO}}_{2}$ activated hard carbons exhibit a larger average interlayer spacing than their untreated counterparts. It should be noted that the sloping capacity reported is defined as the capacity above 100 mV vs. ${\mathrm{Na}}^{+}$/Na. This threshold is inherently arbitrary and may encompass both adsorption‐dominated processes at higher potentials and storage mechanisms, such as pseudo‐intercalation, occurring closer to the 100 mV cut‐off. However, the enlarged interlayer spacing likely improves the accessibility of sodium ions to the carbon structure, thereby facilitating charge storage through adsorption‐related and/or pseudo‐intercalation processes. Although the present data does not allow to isolate the contribution of each structural change, the hard carbons with improved capacity retention all show greater interlayer spacing, higher surface area and fewer C–C single bonds, resulting in increased sloping‐region capacity.

Overall, the hard carbon electrodes demonstrate moderate rate capability in half‐cell configurations, consistent with other hard carbon materials reported in the literature. HC1350_CO2 delivers a reversible capacity of 352 mAh $g^{-1}$ at 0.1 C and retains 219 mAh $g^{-1}$ at 1 C and 183 mAh $g^{-1}$ at 2 C, indicating reasonably fast sodium storage kinetics. The specific capacities at different C‐rates are stated in Table 2. In comparison, many conventional hard carbons typically exhibit capacities of approximately 300–330 mAh $g^{-1}$ at 0.1 C and 80–150 mAh $g^{-1}$ at 2 C [24, 51, 52, 53, 54, 55]. Although specifically engineered hard carbons optimized for either high capacity or high rate capability can reach values of up to ∼390 mAh $g^{-1}$ at 0.1 C and ∼200 mAh $g^{-1}$ at 2 C, the performance of the present material is therefore within the upper range of commonly reported hard carbon anodes [56, 57, 58].

Interestingly, second stage ${\mathrm{CO}}_{2}$ activation resulted in a beneficial effect on coulombic efficiency and cycle life not just for HC1350_CO2, but for all samples. The average CE between cycle 10 and 150 increases with increasing carbonization temperatures for non‐activated samples and further improves after activation of the respective sample, as seen in Table 2. Since this increase in CE and capacity retention is present for both HC900_CO2 and HC1350_CO2 which represents the most disordered and the most ordered sample in terms of TEM electron diffraction, this efficiency increase cannot be attributed to short‐range order. It might, however, be influenced by the reduction of *sp* 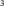 carbon bonds and deficiencies, which is implied by the S band decrease in Raman data for activated samples, as well as the decrease of the C–C peak visible in the XPS data. Although ${\mathrm{CO}}_{2}$ activation reduces ICE due to increased surface reactivity, high‐temperature carbonization samples that underwent activation simultaneously show improved long‐term reversibility, suggesting a transition from aggressive etching to selective structural reorganization.

### 2.3 Impedance Spectroscopy in Pouch Cells and Full Cell Performance

To further characterize these differences in performance, electrochemical impedance spectroscopy was carried out for samples HC900, HC1350, HC900_CO2 and HC1350_CO2. The coated hard carbons were assembled in pouch cells, which deliver higher surface area and generate more reliable impedance data compared to Swagelok cells. To measure the impedance at different state of charge (SOC), a galvanostatic intermittent titration technique (GITT) program was applied. An exemplary GITT potential curve for HC900 is depicted in Figure 8a. Full GITT data is depicted in Figure S13. The total anode resistance was calculated by fitting the resulting semicircles, with exclusion of any Warburg impedance element, as previously discussed in literature [59]. Spectra for all samples are shown in Figures S14 and S15. As seen in Figure 8b, the total anode resistance decreases with increasing SOC for all samples. Furthermore, activation reduces the total resistance for all states of charge when comparing to the non‐activated samples. Figure 8c compares the impedance spectra of the four samples at 95% SOC. It shows sample HC900 having the largest resistance, due to the prominent second semi‐circle often attributed to the charge transfer resistance [60, 61]. This resistance decreases for the later activated sample and further decreases for the samples that have been carbonized at 1350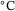. Schutjajew et al. applied distribution of relaxation times (DoRT) to investigate their EIS data and suggested that the ratio change of the semicircles are attributed to different predominating mechanisms of sodium storage [62]. HC900 fit to their description of a mechanism still influenced by an adsorption based mechanism, even at a high SOC, while HC1350 exhibits more pore filling/intercalation behavior. ${\mathrm{CO}}_{2}$ activation presumably leads to a mixture of both processes, with both semi circles having roughly the same size. The lower impedance could be explained by the higher $S_{\mathrm{BET}}$ surface after activation and the overall smaller crystallite size, as well as the general trend of specific capacity (Table 1).

**FIGURE 8:**
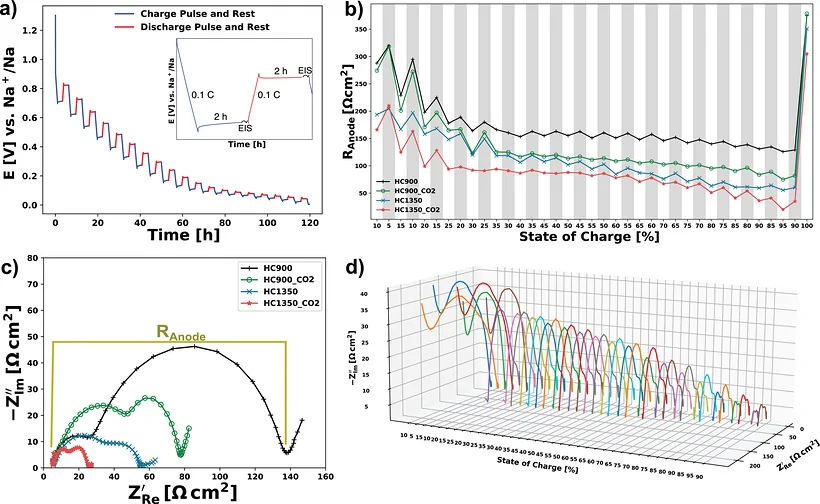
(a) Depiction of GITT program applied to pouch cells. Each sample was charged at 0.1 C for 1 h and rested for two hours before EIS. After each charge, a discharge pulse was applied for 30 min, followed by 2 h of rest and another EIS. Magnification shows points of recording. (b) State of charge vs. total anode resistance. (c) Impedance spectra at 95% SOC. (d) Waterfall plot of all EIS recorded at 5%–95% SOC for sample HC1350_CO2.

Figure 8d shows a waterfall plot of EIS data for sample HC1350_CO2, where a decrease of the second semi‐circle is clearly visible when moving from 5%–95% SOC. However, to gain a deeper insight about the different Na‐storage mechanisms at different SOC for hard carbon generated from pDCPD, further impedance analysis is necessary in the future.

To demonstrate the performance of the most promising sample, a pouch cell with an HC1350_CO2 anode and a balanced NaNiFeMn oxide (424) cathode was assembled in a two‐electrode set‐up and cycled between 3.75 and 1.10 V at 0.3 C. For full cell cycling, the practical anode potential was thereby moved to a maximum of 1.1 V during discharge, which was deemed high enough to ensure utilization of the sloping capacity, but also low enough to avoid SEI decomposition or the use of an impractical potential. The charge and discharge profiles during the formation steps are shown in Figure 9b. Based on the hard carbon mass, the full cell achieves a reversible capacity of 326 mAh $g^{-1}$ at 0.3 C after formation. It shows an initial Coulombic efficiency of 80.3% and an average operating voltage of 3.25 V. The corresponding rate test for the full cell is depicted in Figure 9c. These values could potentially be improved by different cathode materials partnered with HC1350_CO2. Long life cycle stability is promising, as the cell was cycled for 500 cycles at 0.3 C with a capacity retention of 88.1% (calculated from capacity after formation) and an average Coulombic efficiency of 99.81±0.11% for cycles 10–500, indicating good electrochemical stability in carbonate‐based electrolytes. The cycling data is depicted in Figure 9d.

**FIGURE 9:**
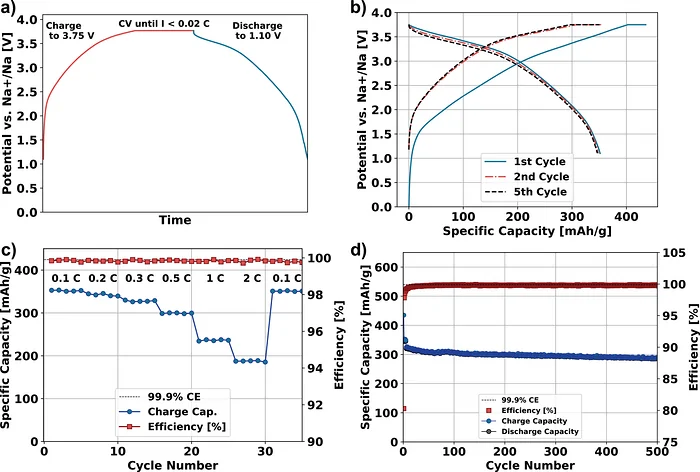
(a) Depiction of cycling program applied to pouch cells for galvanostatic cycling. (b) The first five cycles used for the formation of the cell at 0.1 C. (c) Rate performance of the NaNiFeMnO(424)/HC1350_CO2 full cell after formation. (d) Cycling data of the full cell at 0.3 C. First five cycles correspond to formation at 0.1 C. Specific capacity calculated for anode mass.

Hard carbon anodes frequently suffer from capacity fade in conventional carbonate based electrolytes due to continuous solid electrolyte interphase (SEI) growth and electrode degradation, often resulting in capacity retentions of only 70%–80% after 400–500 cycles [23, 25, 26, 27, 28, 29]. Consequently, numerous recent studies have focused on improving the electrolyte composition or the hard carbon structure to enhance interfacial stability and mitigate irreversible sodium consumption. Representative examples include the use of ether based electrolytes, tailored hard carbon microstructure, and electrolyte additives [24, 63, 64, 65, 66, 67, 68, 69]. A detailed comparison of the electrochemical performance reported in these studies is provided in Table S3. In comparison, the hard carbon developed in this work retains 88.1% of its capacity after 500 cycles in a conventional carbonate based electrolyte, demonstrating competitive long term stability without relying on alternative electrolyte chemistry or specialized electrolyte additives.

## 3 Conclusions

In this work, we have synthesized poly(dicyclopentadiene) based hard carbon and investigated the influence of second step ${\mathrm{CO}}_{2}$ activation on the material. Thereby presenting an easy‐to‐implement approach for synthesizing a carbon‐based anode material for sodium–ion batteries from a readily available raw material. Furthermore, we highlight how variations in the carbon structure influence electrochemical performance. With TEM, $S_{\mathrm{BET}}$ surface data and cyclic voltammetry, we demonstrate that the first step carbonization temperature influences the impact of ${\mathrm{CO}}_{2}$ activation on the morphology of the hard carbon. Lower first step temperature resulted in a significant surface increase and high local disorder, while the sample carbonized at 1350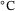 prior to activation, exhibited the highest short‐range order of all samples and only minor surface increase. The chemical impact of ${\mathrm{CO}}_{2}$ treatment was investigated with elemental analysis, Raman and XPS measurements. The data showed a decrease in carbon content after activation and suggests a removal of C–C *sp* 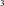 bonds. Structural analysis with SAXS and WAXS data showed an increase in nano pore size with increased carbonization temperature and a higher interlayer distance after consecutive ${\mathrm{CO}}_{2}$ treatment. Electrochemical testing further showed an increase in coulombic efficiency, capacity retention and rate performance after ${\mathrm{CO}}_{2}$ activation. These findings indicate that high‐temperature ${\mathrm{CO}}_{2}$ activation promotes selective structural reorganization rather than aggressive etching, enabling improved long‐term reversibility despite lower ICE. The best performing hard carbon, carbonized at 1350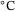 and activated with ${\mathrm{CO}}_{2}$, was assembled in a pouch cell with NaNiFeMn(424) as cathode active material. The negative electrode showed a capacity of 352.2 mAh $g^{-1}$ at 0.1 C and the pouch cell exhibited promising capacity retention, with 88.1% after 500 cycles at 0.3 C making pDCPD based hard carbon a promising material for Na‐ion batteries that focus on long cycle life. In addition, the material can be synthesized via a straightforward route without the need for high‐pressure processing or atomic layer deposition and is compatible with commonly used carbonate‐based electrolytes, supporting its potential for scalable application.

## 4 Experimental Section

### 4.1 Materials and Methods

Dicyclopentadiene (DCPD, Aldrich), Pluronic L‐121 (poly(ethylene glycol)‐block‐poly(propylene glycol)‐block‐poly(ethylene glycol, Aldrich), the initiator ($H_{2}\mathrm{IMes}$)(${\mathrm{PCy}}_{3}$)${\mathrm{Cl}}_{2}\mathrm{Ru}$(3‐phenyl‐indenylid‐1‐ene) (M202, Umicore, $H_{2}\mathrm{IMes}$ = N,N‐bis(mesityl) 4,5‐dihydroimidazol‐2‐yl), ${\mathrm{PCy}}_{3}$ = tricyclohexylphosphine)) and toluene (p.a. Aldrich) were used as received.

Morphology was investigated using scanning electron microscopy (SEM). SEM images were taken on a Sigma 560VP Gemini 1 Carl Zeiss microscope equipped with an Ultim Extreme energy‐dispersive X‐ray spectroscopy (EDX) detector from Oxfrord Instruments. The WAXS diffraction data of the hard carbon samples was obtained using a XRDynamik 500 powder X‐ray diffractometer (Anton Paar). The instrument was applied in Bragg–Brentano geometry and was operated using Cu‐Kα radiation (λ=0.15418 nm). Diffraction angles were measured from 5 to 100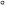 2θ, with a step size of 0.01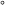 2θ and 200 s per step. Data evaluation was performed using the X'Pert Highscore Plus software. Small‐angle X‐ray scattering data was collected on all samples using an SAXSpoint 2.0 instrument from Anton Paar. Scattering data was collected on an Eiger 1M detector (Dectris, Switzerland) positioned 113 and 552 mm from the sample, using a microfocused, monochromatized X‐ray source Cu Kα (λ=0.154 nm). The samples were held in the beam between two pieces of Scotch Magic tape, and the resulting data was processed using the DAWN software package, using standardized data correction procedures. The corrected data were fit using Sasfit. Elemental analysis was performed with an Elementar Vario MICRO cube. Raman measurements were performed in a capillary using a Raman Station 400F (Perkin Elmer, Waltham, MA, USA) with a built‐in 350 mW laser operating at 785 nm. The TEM measurements were carried out on a JEM‐2200FS (JEOL) instrument in brightfield mode operated at 200 kV and equipped with a Gatan CCD camera. For the X‐ray photoelectron spectroscopy (XPS) measurements, a Specs Phoibos 150 CCD‐9 spectrometer was used with an Al‐Kα source (1486.71 eV) in a vacuum of $<10^{-8}$ mbar. Measurements were carried out with and without the use of a flood gun. Particle size distribution was determined by static light scattering (SLS) using a FRITSCH Analysette 22 Nanotec plus with “Frauenhofer” method. To aid dispersion, a drop of the surfactant FRITSCH Dusazin 901 was added to the powder or suspension and mixed with a spatula until a paste‐like consistency was achieved. Subsequently, 1 g of sample was dispersed in 20–30 mL of deionized water and treated in an ultrasonic bath for 10 min prior to the SLS measurement. The specific surface areas were determined by the Brunauer–Emmett–Teller (BET) method in accordance with ISO 9277:2010(E) standards with $N_{2}$ and Ar gas.

### 4.2 Synthesis

Synthesis of hard carbon: In a three‐necked 250 mL flask, 64 g of dicyclopentadiene (DCPD), 2560 mg of poly(ethylene glycol)‐block‐poly(propylene glycol)‐block‐poly(ethylene glycol) (Pluronic L‐121, equivalent to 4 wt.% of DCPD), and 400 μL of toluene were combined. The mixture was stirred using an overhead stirrer at a speed of 400 rpm. To achieve an emulsion containing 80% water, 256 mL of deionized water were added drop wise with continuous stirring. After the complete addition of water, the mixture was further stirred for 1 h until a uniform emulsion formed. Next, a solution of 12 mg dichloro[1,3‐bis(2,4,6‐trimethylphenyl)‐2‐imidazolidinylidene](3‐phenyl‐1H‐inden‐1‐ylidene)(tricyclohexylphosphine)ruthenium(II) (referred to as M202) ROMP initiator in toluene (1 mL) was introduced into the emulsion, and the mixture was stirred for 30 s at a speed of 800 rpm. Subsequently, the emulsion was transferred into glass vials acting as molds and cured at 80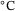 for 4 h. The resulting polymers were removed from their molds and subjected to a washing step in an acetone bath. The cylindrical monoliths were then oxidized in an oven operating at 40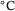 for a period of 7 days, followed by carbonization in a nitrogen atmosphere for 2 h at temperatures of either 900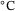, 1050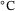, 1200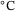 or 1350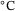, with a ramp‐up rate of 10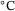 per minute and a ramp‐down rate of 10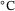 per minute. Activated samples were subsequently held at 900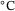 for 1 h and the gas flow was switched to ${\mathrm{CO}}_{2}$ with a flow rate of 80 mL $\min^{-1}$. Non‐activated samples underwent the second plateau at 900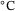 under $N_{2}$ atmosphere. Afterward, the gas flow was switched back to $N_{2}$ and the samples were cooled to room temperature at 10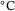 per minute.

### 4.3 Electrochemical Characterization

Electrode preparation and electrochemical measurements: The hard carbon samples were pre‐ground with a mortar and then fine‐ground with a WC planetary ball mill at 500 rpm for 2 min. The powder was sieved with a 20 μm net in a sieving tower, with a pulse of 5 Hz for 10 min. For electrode preparation, a slurry with 94 w% hard carbon, 2 w% styrene–butadiene rubber (SBR) binder, 2 w% Na‐carboxy methylcellulose (NaCMC) and 1 w% Super C65 was mixed in water, stirred overnight and coated on Al foil with a doctor blade. The mass loading of hard carbon was 3.0 to 3.3 mg ${\mathrm{cm}}^{-2}$. The anodes were further densified via a calendar to 90% of former thickness. As electrolyte, 1 M ${\mathrm{NaClO}}_{4}$ in ethylene carbonate: propylene carbonate: dimethyl carbonate (EC:PC:DMC/1:1:1 in volume) with 2 w% vinylene carbonate (VC) and 1 w% dimethoxyethane (DME) was used. Glass fibre was used as a separator. Sodium metal foil was used as counter and reference electrode in half cell testing in a Swagelok cell set up. All cell assemblies were performed in an Ar‐filled glove box. For galvanostatic cycling a Biologic MPG2 was used. All cells were formed at 0.1 C for 5 cycles with 1.6 V and 5 mV as cut‐off potential, before various C‐rates were applied. Cyclic voltammetry (CV) was performed from 1.6 V to 5 mV with a scan rate of 30 μV $s^{-1}$. For all rate performance tests in full‐cells and half‐cells, the current density was calculated from the reversible capacity after 5 cycles at 0.1 C. For sodium‐ion full cells in a pouch cell set‐up, NaNiFeMn (424) oxide was used as cathode material. A slurry with 94 w% active material, 3 w% PVDF and 3 w% Super C65 was stirred in NMP overnight, sieved with a 45 μm sieve and coated on Al foil with a doctor blade. The cathodes were further densified via a calendar to 70% of former thickness. The cathodes were balanced to reach 97% of the anodes capacity at complete charge for galvanostatic cycling and overbalanced to 130% for impedance testing. For galvanostatic cycling, full cells were cycled at different C‐rates from 3.75 to 1.10 V, with a constant voltage step in the charging step at 3.75 V that was applied until a current <C/50 was reached. For the impedance pouch cells, a sodium foil reference was placed opposite to the negative electrode. A Biologic VMP3 was used for electrochemical impedance spectroscopy (EIS). After 5 cycles at 0.1 C and 30 cycles at 0.3 C, each pouch cell was charged with a GITT pulse of 0.1 C for 1 h, followed by a 2 h OCV period. EIS was performed from 100 kHz to 10 mHz with an amplitude of 10 mV and the cells were subsequently discharged at 0.1 C for 30 min followed by another 2 h OCV period and EIS. These steps were continued until a state of charge (SOC) of 100% was reached and 5 mV were held for 1 h to guarantee full charge.

## Author Contributions

David Schuster: conceptualization, visualization, methodology, writing – original draft. Glen J. Smales: measurements and data analysis, Max Schmallegger: measurements and data analysis, Julian M Rosalie: measurements and data analysis, Konstantin Kraushaar: measurements and data analysis, Andreas Lißner: measurements and data analysis, Julia Bohdal: measurements and data analysis, Pia Winter: measurements and data analysis, Bernd Fuchsbichler: supervision, Stefan Koller: supervision, Sebastijan Kovačič: measurements and data analysis, review, editing, Christian Slugovc: supervision, review, editing.

## Conflicts of Interest

The authors declare the following financial interests/personal relationships which may be considered as potential competing interests: David Schuster, Bernd Fuchsbichler and Christian Slugovc are inventors on a pending patent application submitted by VARTA Innovation GmbH and TU Graz regarding polydicyclopentadien based hard carbon as an anode for sodium‐ion batteries. (application pending) The remaining authors declare no competing interests.

## Supporting information


**Supporting File**: advs78311‐sup‐0001‐SuppMat.docx.

## Data Availability

The data that support the findings of this study are available from the corresponding author upon reasonable request.
