# Supplementary material for: Activation‐Induced Turbostratic Ordering in pDCPD‐Derived Hard Carbon Enables Stable Carbonate‐Based Sodium‐Ion Storage
Source: Adv Sci (Weinh). 2026 Oct 18:e78311. Online ahead of print. doi: 10.1002/advs.78311 (PMC13676995; doi:10.1002/advs.78311)
Supplement: Supplementary file 1 [file ADVS-9999-e78311-s001.docx]

Supporting Information for:

Activation-Induced Turbostratic Ordering in pDCPD-Derived Hard Carbon Enables Stable Carbonate-Based Sodium-Ion Storage

David Schuster ^a,b,c,^*, Glen J. Smales ^d^, Max Schmallegger ^e^, Julian M Rosalie ^f^, Konstantin Kraushaar ^g^, Andreas Lißner ^h^, Julia Bohdal ^c^, Pia Winter^c,d^ Bernd Fuchsbichler ^c,^*, Stefan Koller ^c^, Sebastijan Kovačič ^I,j^, Christian Slugovc ^a,b^

^a^ Christian Doppler Laboratory for Organocatalysis in Polymerization, Graz University of Technology, Stremayrgasse 9/V, A 8010 Graz, Austria

^b^ Institute for Chemistry and Technology of Materials, Graz University of Technology, Stremayrgasse 9/V, 8010 Graz, Austria

^c^ VARTA Innovation GmbH, Am Innovationspark 10, 8020 Graz, Austria

^d^ Institute of Inorganic Chemistry, Graz University of Technology, Stremayrgasse 9/V, 8010 Graz, Austria

^e^ Institute for Physical and Theoretical Chemistry, Graz University of Technology, Stremayrgasse 9/II, A 8010 Graz, Austria

^f^ Federal Institute of Materials Research and Testing (BAM), Unter der Eichen 87, Berlin 12205, Germany

^g^ Institute of Inorganic Chemistry, TU Bergakademie Freiberg, Leipziger Str. 29, 09599 Freiberg Germany

^h^ Institut of Physical Chemistry, TU Bergakademie Freiberg, Leipziger Str. 29, 09599 Freiberg, Germany

^i^ Catalysis and Organic Synthesis research group, Faculty of Chemistry and Chemical Engineering, University of Maribor, Smetanova 17, SI-2000 Maribor, Slovenia

^j^ Department of Inorganic Chemistry and Technology, National Institute of Chemistry, Hajdrihova 19, SI-1001 Ljubljana, Slovenia

*corresponding author; email: schuster@student.tugraz.at, bernd.fuchsbichler@varta-ag.com

**Particle size determination**

Particle size distribution was determined by static light scattering (SLS) using a *FRITSCH Analysette 22 Nanotec plus* with “Frauenhofer” method. To aid dispersion, a drop of the surfactant *FRITSCH Dusazin 901* was added to the powder or suspension and mixed with a spatula until a paste-like consistency was achieved. Subsequently, 1 g of sample was dispersed in 20–30 mL of deionized water and treated in an ultrasonic bath for 10 minutes prior to the SLS measurement.

Figure S1: Particle size distribution of HC900, HC900_CO2, HC1050 and HC1050_CO2 determined via SLS.

Figure S2: Particle size distribution of HC1200, HC1200_CO2, HC1350 and HC1350_CO2 determined via SLS.

**SEM Measurements**

Scanning electron microscopy (SEM) measurements were carried out on a Carl Zeiss Sigma 560VP Gemini 1 microscope. Secondary electron images were recorded at an accelerating voltage of 2 kV and a working distance of 7.8 mm.

**10 μm**

**100 μm**

**10 μm**

Figure S3: Exemplary SEM Image (5k magnification) of HC1350_CO2 before (top) and after (bottom) ball milling.

**TEM**

The TEM measurements were carried out using a JEM-2200FS (JEOL) instrument operated in bright-field mode at an accelerating voltage of 200 kV. The instrument was equipped with a Gatan CCD camera.

Figure S4: TEM images of HC1350_CO2

**N_2_ BET**

Porosity properties were evaluated by nitrogen sorption at 77 K using a Micromeritics TriStar II 3020 instrument. Before measurement, samples were degassed under high-purity nitrogen using a two-stage protocol: 90 °C for 60 min, followed by 160 °C for 240 min, with heating rates of 10 °C min^-1^. Surface areas were determined by the Brunauer–Emmett–Teller (BET) method in accordance with ISO 9277:2010(E), using adsorption data in the P/P_0_ = 0.06–0.30 range.

Figure S5: N_2_ adsorption− desorption isotherms of different hard carbon samples for activated and not activated pairs.

**Ar BET**

Argon isothermal measurements were carried out on an iQ3 gas analyser (Quantachrome Instruments) at 87 K, respectively. Prior to the measurements, the sample was outgassed at 150 °C for 16 hours.

Figure S5b: Ar adsorption− desorption isotherms of different hard carbon samples for activated and not activated pairs.

**XRD**

The diffraction data of the hard carbon samples were collected using an XRDynamik 500 powder X-ray diffractometer (Anton Paar). The instrument was operated in Bragg–Brentano geometry with Cu Kα radiation (λ = 0.15418 nm). Diffraction angles were recorded in the range of 5–100° 2θ with a step size of 0.01° 2θ and a counting time of 200 s per step. Data analysis was performed using the X’Pert Highscore Plus software. The background was subtracted with a spline function through the background region from 0-10 2Theta in OriginLab software. Additionally, a second set of measurements was performed with an internal Si reference for 002 peak tracking, as seen in figure S6b.

Figure S6: XRD background correction for all hard carbon samples.

Figure S6b: XRD data for all hard carbon samples with added Si reference for peak tracking. Dotted line added at 23 [2°Theta].

**Small-angle X-ray scattering (SAXS)**

Small-angle X-ray scattering (SAXS) data were collected for all samples using a SAXSpoint 2.0 instrument from Anton Paar. The scattering data were recorded with an Eiger 1M detector (Dectris, Switzerland) positioned at distances of 113 mm and 552 mm from the sample. A micro-focused, monochromatized Cu Kα radiation source (λ = 0.154 nm) was used for the measurements. The samples were mounted between two strips of Scotch Magic Tape during data acquisition. The resulting data were processed using the DAWN software package applying standard data-correction procedures and subsequently analyzed with Sasfit.

Figure S7: Example fit of HC900. Four contributions were fit to the data, background, meso pores, nano pores and interlayer distance.

**Raman Spectroscopy**

The Raman measurements were carried out on an optical plate using a Raman Station 400F (Perkin Elmer, Waltham, MA, USA). The instrument was equipped with a built-in 350 mW laser operating at 785 nm. The data was fit with five “Pseudo Voigt” distributions. The first guess was started with distributions around 1100-1250, 1250-1350, 1400-1550, 1570-1620 and 1580-1630 cm^-1^.

Figure S8: Deconvoluted Raman spectra of HC900, HC900_CO2, HC1050 and HC1050_CO2

Figure S9: Deconvoluted Raman spectra of HC1200, HC1200_CO2, HC1350 and HC1350_CO2

**X-ray photoelectron spectroscopy  (XPS)**

For the X-ray photoelectron spectroscopy (XPS) measurements, a Specs Phoibos 150 CCD-9 spectrometer was used with an Al-Kα source (1486.71 eV) in a vacuum of <10^-8^ mbar. Measurements were carried out with and without the use of a flood gun. XPS data was fit according to literature for conductive carbon samples.^1^

Figure S10: C1s XPS spectra of HC900 and HC900_CO2. Measurement with flood-gun "wfg" and no floog-gun "nfg".

Figure S11: C1s XPS spectra of HC1350 and HC1350_CO2. Measurement with flood-gun "wfg" and no floog-gun "nfg".

Table S1: Quantitative analysis of samples, with mean value of nfg and wfg measurements

| Sample | Atomic % C1s | Atomic % O1s |
| --- | --- | --- |
| HC900 | 95.5 | 4.5 |
| HC900_CO2 | 96.0 | 4.0 |
| HC1350 | 93.9 | 6.1 |
| HC1350_CO2 | 96.5 | 3.5 |

| Sample | C=C | C-C | C-O | C=O | O-C=O | π to π* |
| --- | --- | --- | --- | --- | --- | --- |
| HC900 | 79.50 ± 1.25 | 9.36 ± 0.10 | 3.50 ± 0.12 | 2.77 ± 0.17 | 2.33 ± 0.11 | 2.55 ± 0.64 |
| HC900_CO2 | 85.76 ± 0.45 | 1.96 ± 0.42 | 2.64 ± 0.18 | 2.39 ± 0.35 | 2.53 ± 0.52 | 5.08 ± 0.87 |
| HC1350 | 80.24± 0.13 | 6.70± 0.00 | 3.68±0.07 | 2.38 ± 0.06 | 2.08± 0.04 | 4.92± 0.03 |
| HC1350_CO2 | 82.90 ± 2.32 | 1.98 ± 0.16 | 3.83 ± 0.01 | 3.09 ± 0.43 | 2.91 ± 0.28 | 5.35 ± 0.10 |

*Table S2: Quantitative analysis o peaks for all samples, with mean value of nfg and wfg measurements*

Figure S12: XPS survey scan for HC1350 and HC1350_CO2 (left) and HC900 and HC900_CO2 (right).

**Galvanostatic Intermittent Titration Technique (GITT) Raw Data**

Figure S13: GITT Raw data of pouch cells with reference electrode containing HC900, HC900_CO2, HC1350 or HC1350_CO2 as negative electrode.

**Electrochemical Impedance Spectroscopy (EIS) Raw Data**

Figure S14: Waterfall plot showing EIS data corresponding to GITT measurements for pouch cells containing HC900 (above) and HC900_CO2 (below).

Figure S15: Waterfall plot showing EIS data corresponding to GITT measurements for pouch cells containing HC1350 (above) and HC1350_CO2 (below).

**Comparison with recent carbonate-based Na-ion cells**

**Table S3: Comparison/list of recent hard carbon based Na-ion cells that use carbonate electrolyte.**

| **Reference** | **ICE (%)** | **Cap. retention (%)** | **Cycles** | **Remarks** |
| --- | --- | --- | --- | --- |
| **Sun et al. (2024)^2^** | **96 (ether)** | **85 (ether)** | **200** | **Ether electrolyte substantially outperformed carbonate electrolyte. No exact values for carbonate system stated, but significantly lower than ether-based configuration.** |
| **Lu et al. (2024)^3^** | **98 (ether)** | **79 (ether)** | **500** | **Highest performance obtained using a pure ether electrolyte. No exact values for carbonate system stated, but significantly lower than ether-based configuration.** |
| **Du et al. (2024)^4^** | **90** | **80** | **300** | **Enhanced pseudo-graphitic ordering reduced intrinsic defects, leading to improved ICE and stable cycling.** |
| **Liu et al. (2024)^5^** | **90** | **81** | **300** | **Defect repair through trace P doping improved the balance between high ICE and enhanced sodium-storage capacity.** |
| **Liu et al. (2025)^6^** | **85** | **79** | **200** | **Hard carbon recovered from end-of-life batteries.** |
| **Büchele et al. (2025)^7^** | **85** | **79** | **300** | **Hard carbon used for evaluating Prussian blue analogues** |
| **Li et al. (2024)^8^** | **84** | **89** | **200** | **Cross-linking with glucose reduced defect density while maintaining a porous structure, improving Na⁺ storage.** |
| **Meng et al. (2025)^9^** | **89** | **90** | **400** | **Improved SEI stability through ionic liquid electrolyte additive.** |
| **Chen et al. (2022)^10^** | **44** | **86** | **100** | **Biomass-derived porous hard-carbon spheres with a microporous structure exhibited improved cycling stability and sodium-storage performance.** |
| **Beda et al (2021)^11^** | **92** | **80** | **300** | **Low specific surface area and low ultramicroporosity enabled a high ICE (92%) and high volumetric capacity.** |
| **This work** | **80** | **88** | **500** | **Second-stage CO_2_ activation allows the use of carbonate-based electrolytes without unconventional additives** |

**References**

(1) Biesinger, M. C. Accessing the Robustness of Adventitious Carbon for Charge Referencing (Correction) Purposes in XPS Analysis: Insights from a Multi-User Facility Data Review. *Appl. Surf. Sci.* 2022, *597* (May). https://doi.org/10.1016/j.apsusc.2022.153681.

(2) Sun, W.; et al. Molecular Engineering Enabling High Initial Coulombic Efficiency and Robust Solid Electrolyte Interphase for Hard Carbon in Sodium-Ion Batteries. *Angew. Chem. Int. Ed.* 2024, *63*. https://doi.org/10.1002/anie.202318960.

(3) Lu, Z.; Yang, H.; Guo, Y.; et al. Consummating Ion Desolvation in Hard Carbon Anodes for Reversible Sodium Storage. *Nat. Commun.* 2024, *15*, 3497. https://doi.org/10.1038/s41467-024-47522-y.

(4) Du, Y.; Qiu, Y.; Zhuang, R.; Jing, X.; Liu, D.; Peng, X.; Yan, L.; Xu, F. Superior Initial Coulombic Efficiency and Areal Capacity of Hard Carbon Anode Enabled by Graphite-Assisted Carbonization for Sodium-Ion Battery. *Carbon* 2024, *221*, 118929. https://doi.org/10.1016/j.carbon.2024.118929.

(5) Liu, Z.-G.; Zhao, J.; Yao, H.; et al. P-Doped Spherical Hard Carbon with High Initial Coulombic Efficiency and Enhanced Capacity for Sodium Ion Batteries. *Chem. Sci.* 2024, *15* (22), 8478–8487. https://doi.org/10.1039/D4SC01395F.

(6) Liu, X.; et al. Sustainable Recovery and Reuse of Hard Carbon from Scrap and End-of-Life Sodium-Ion Batteries. *Adv. Energy Mater.* 2025. https://doi.org/10.1002/aenm.202405894.

(7) Büchele, S.; Mereacre, V.; Bohn, N.; et al. Sodium Manganese Hexacyanoferrate: Characterization as Sodium-Ion Battery Cathode Material, Full Cell Cycling with Hard Carbon and Post-Mortem Analyses. *Batteries Supercaps* 2025, *8* (9). https://doi.org/10.1002/batt.202500015.

(8) Li, Y.; et al. Design of Cross-Linked Hard Carbon with High Initial Coulombic Efficiency for Sodium-Ion Batteries Anode. *Chem. Phys. Lett.* 2024, *857*, 142717. https://doi.org/10.1016/j.cplett.2024.141651

(9) Meng, Y.; et al. Improvement of Interphase Stability of Hard Carbon for Sodium-Ion Battery by Ionic Liquid Additives. *Batteries* 2025, *11* (3), 102. https://doi.org/10.3390/batteries11030102

(10) Chen, S.; Tang, K.; Song, F.; Liu, Z.; Zhang, N.; Lan, S.; Xie, X.; Wu, Z. Porous Hard Carbon Spheres Derived from Biomass for High-Performance Sodium/Potassium-Ion Batteries. *Nanotechnology* 2022, *33* (5), 055401. https://doi.org/10.1088/1361-6528/ac317d

(11) Beda, A.; Rabuel, F.; Morcrette, M.; Knopf, S.; Taberna, P.-L.; Simon, P.; Ghimbeu, C. M. Hard Carbon Key Properties Allow for the Achievement of High Coulombic Efficiency and High Volumetric Capacity in Na-Ion Batteries. *J. Mater. Chem. A* 2021, *9* (3), 1743–1758. https://doi.org/10.1039/D0TA07687B
